# Supplementary material for: The gender gap in Ph.D. entrepreneurship: How do students perceive the academic environment?
Source: PLoS One. 2022 Apr 7;17(4):e0261495. doi: 10.1371/journal.pone.0261495 (PMC8989309; doi:10.1371/journal.pone.0261495)
Supplement: S1 Questionnaire — (DOCX) [file pone.0261495.s002.docx]

| **QUESTIONNAIRE SELF-ASSESSMENT PhD STUDENTS** |
| --- |
| **Personal characteristics** |
| Year of birth |
| Gender (male=1) |
| Citizenship (foreign=1) |
| Province (NUTS3) where you live |
| Does at least one of your parents hold a university degree (yes=1)? |
| Is at least one of your parents a university lecturer (yes=1)? |
| Is at least one of your parents an entrepreneur (yes=1)? |
| **PhD programme** |
| Have you completed your PhD? |
| If yes, when (year) |
| If not, in what year of study are you |
| Institution |
| Department |
| PhD programme |
| Principal subject |
| *Mathematics & Computer Science* |
| *Physics* |
| *Chemistry* |
| *Geology* |
| *Biology* |
| *Medicine* |
| *Agriculture & Veterinary* |
| *Civil Engineering & Architecture* |
| *Engineering* |
| *Humanities* |
| *Sociology, philosophy and psychology* |
| *Law* |
| *Economics and Statistics* |
| *Political Sciences* |
| Your opinion about the PhD programme (1~6=highest): |
| *Competence of the supervisor* |
| *Time dedicated to research activity* |
| *Availability of equipment and res. Infrastructure* |
| *Availability of financial resources* |
| *Degree of independence/autonomy of res. Subjects* |
| *Extension/quality of the international res. Network* |
| *Degree of international experience* |
| *Quality of the research team* |
| *Access to labour market* |
| My PhD scholarship funded by the private sector (yes=1) |
| **PhD network** |
| Have you spent a visiting period abroad? (yes=1) |
| *n. of months* |
| *Institution* |
| Relevance of the following factors concerning your visiting period (1~6=highest): |
| *Availability of time to perform research tasks* |
| *International experience* |
| *Availability of research funding* |
| *Access to research equipment/facilities* |
| *Autonomy/independence* |
| *International research network* |
| *Relations with the supervisor abroad* |
| *Relations with other members of the research team* |
| *Reputation of the supervisor abroad* |
| *Reputation of the foreign institution* |
| *Improvement of language skills* |
| My PhD research based on collaboration with companies (yes=1) |
| My PhD research influenced by business needs (1~6=highest) |
| My PhD research is oriented towards immediate application of results in a business environment (1~6=highest) |
| Share of my PhD research dedicated to basic research |
| Did you use private facilities/laboratories during your study period? |
| **First degree** |
| Institution |
| University course |
| Year of graduation |
| Final mark |
| Did you start your PhD immediately after the first degree? |
| *If not, what did you do during the gap period?* |
| **Output of the PhD** |
| Did you apply for patents? (yes=1) |
| *If yes, how many?* |
| *In which office? (UIB, EPO, USPTO, JPO)* |
| *In how many of these patents there is at least a company?* |
| *How many of these patents have been licensed to companies?* |
| How many papers have been accepted for publication within two years from graduation on national journals? |
| And after? |
| How many papers have been accepted for publication within two years from graduation on international journals? |
| And after? |
| **PhD supervisor** |
| What is your opinion concerning the international reputation of your PhD supervisor? (1~6=highest) |
| To what extent the inputs of your supervisor have determined the succesful completion of your thesis? (1~6=highest) |
| How many other international peers have influenced your thesis? |
| **Employment condition** |
| Do you work at the moment? (yes=1) |
| *If not, what is your current situation?* |
| *I have applied for work* |
| *I am looking for work in academia* |
| *I am not working because of personal reasons* |
| *I am doing some training of I am attending a course* |
| Have you established a business start-up (yes=1)? |
| *If so, is the start-up is still active (yes=1)?* |
| *Number of partners* |
| *Number of persons employed in the startup created by the student* |
| *Number of partners with an academic background* |
| Have you abandoned the idea of establishing a business start-up (yes=1)? |
| Current position |
| *The student is currently employed in academia/PRC (yes=1)* |
| *Indicate your position* |
| *The student is currently employed as an autonomous worker (yes=1)* |
| *Indicate your position* |
| *The student is currently employed (yes=1)* |
| *Indicate your position* |
| If you are an entrepreneur, please indicate the type of business you are involved in: |
| *Academic spin-off* |
| *Corporate spin-off* |
| *Start-up* |
| *Other* |
| *Age of the enterprise (years)* |
| *Was the start-up incubated (ys=1)?* |
| *Where?* |
| *Where is the start-up located?* |
| *Did the entrepreneurial venture benefited from public funding?* |
| *If yes, from the ERDF* |
| *If yes, from the ESF* |
| *If yes, from other sources* |
| Did you attend a course on Entrepreneurship? (yes=1) |
| *Yes, after the PhD* |
| *Yes, during the PhD* |
| *Yes, between the frist degree and the PhD* |
| *Yes, as part of the first degree programme* |
| *Yes, other* |
| Did the entrepreneurial venture benefited from private funding? |
| *No* |
| *Yes, loans from friends and family* |
| *Yes, loans from banks* |
| *Yes, from business angels* |
| *Yes, from a venture capital fund* |
| *Yes, other* |
| How much are you satisfied of your current employment condition? (1~6=highest) |
| Would you be interested in creating a start-up regardless of your current employment condition? (1~6=highest) |
| Would you be interested in creating a start-up which is based on your academic background? (1~6=highest) |
| Have you ever discussed a business idea with your supervisor? (yes=1) |
| **Entrepreneurial Opportunity - Risk taking - Opportunity recognition** |
| Is "intuition" important in starting a business? |
| How important are these factors in an hypothetical startup creation? |
| *Funding from venture capitalists* |
| *Funding from banks* |
| *Funding from friends* |
| *Initial planning and strategies* |
| *Learning in progress and strategy revision* |
| *Capacity to exploit opportunities* |
| Do you think you are capable of starting a company (1~6=absolutely yes)? |
| In starting a company, how important it is to rely on well-tested and reliable products (1~6=absolutely important)? |
| Do you think that being a "pioneer" is more an advantage or a risk (1~6=absolutely a risk)? |
| To what extent are you inclined to invest in technologies/projects/products whose marketability is uncertain (1~6=absolutely inclined)? |
| Are you more inclined to invest in low-risk projects with normal returns, or in high-risk projects with high returns (1~6=absolutely uncertain)? |
| Are you more an easygoing or a competitive person (1~6=absolutely competitive)? |
| **Drivers and obstacles to business start-up** |
| Students' opinion about the relevance of drivers to entrepreneurship (1~6=highest) |
| *Public funding* |
| *Private funding* |
| *Bank loans* |
| *Financial partners* |
| *Industrial partners* |
| *Field experts* |
| *Patent portfolios* |
| *Scientific support* |
| *Startup assistance* |
| *Business plan* |
| *Market analysis* |
| *Incubation* |
| *Design* |
| *Legal support* |
| *Business competition schemes* |
| Students' opinion about the relevance of obstacles to entrepreneurship (1~6=highest) |
| *Unclear (lack of) academic rules and guidelines on startup creation* |
| *Difficulties in raising financial resources* |
| *Difficulties in finding appropriate know-how and scientific competencies* |
| *Difficulties in finding appropriate managerial competencies* |
| *Difficulties in finding appropriate equipment and capital goods* |
| *Difficulties in finding information on markets* |
| *Lack of networks* |
| *Low risk attitude* |
| *Low entrepreneurial attitude of the supervisor* |
| *Lack of suitable partners* |
| *Excessive bureaucracy* |
| *Necessity of authorisations* |
| **Academic entrepreneurship** |
| The student agrees that in the home university (1~6=highest): |
| *there is a favourable environment to startup creation* |
| *there is a favourable environment to U-I interaction* |
| *entrepreneurship is a central mission* |
| *there is support to patenting and innovation* |
| *there is dedicated strategy to technology transfer* |
| *U-I collaboration is important* |
| *teaching is well connected to research* |
| *there are training courses for entrepreneurs* |
| *there is professional support to potential entrepreneurs* |
| In terms of research valorisation, what is the extent of support that your university offers (1~6=highest): |
| *Along the whole entrepreneurial process, not just in the definition of the business idea* |
| *In the patenting process* |
| *In the identification of business opportunities* |
| *In the preparation of a business plan* |
| *In the creation of an academic spin-off* |
| *In the creation of a start-up* |
| *In finding external funding* |
| *In licensing activities* |
| *In the negotiation with external partners* |
| With reference to research internationalisation, I believe that my university (1~6=highest): |
| *Is focused on research internationalisation* |
| *Is well connected at the international level for R&D activities* |
| *Is well connected with other universities for teaching programmes* |
| *Offers the opportunity to get a PhD in collaboration with foreign institutions* |
| Did your university establish any business-plan competition? (yes=1) |
| Do you find appropriate the incentive system of your university in encouraging research valorisation and entrepreneurship (1~6=absolutely yes)? |
