# Supplementary material for: The gender gap in Ph.D. entrepreneurship: How do students perceive the academic environment?
Source: PLoS One. 2022 Apr 7;17(4):e0261495. doi: 10.1371/journal.pone.0261495 (PMC8989309; doi:10.1371/journal.pone.0261495)
Supplement: Attachment — Submitted filename: Response Plos One.docx [file pone.0261495.s003.docx]

**RESPONSE TO REVIEWERS**

Reviewer #1: This paper is interesting and well written. The topic is very well focused, and the possible applications of the conclusions could be valuable. The statistic analysis is also interesting, but I would like to use a deeper analysis with a complementary method, for example, an ANOVA comparing results. If possible, I recommend completing the paper. Besides, the complements of qualitative information will substantially improve if more explicative information is provided.

RESPONSE: Thank you for the encouraging words. We did not run the ANOVA as we analyse just two groups (male vs. female). However, as part of our efforts to respond to Reviewer #2, we added an econometric analysis to the results. We hope that this responds also to your request to complete the analysis. We also added better explicative information.

Reviewer #2: Novo-Cortí & Muscio (2021) examine gender gaps in Ph.D. entrepreneurship through a 2014-15 survey of Italian doctoral students. They make comparisons between male and female students’ perceptions about successful entrepreneurship and draw connections to the support offered by their universities. Although their survey provides important data on doctoral students’ perceptions of entrepreneurship, more detail is necessary in the paper and the analyses performed to reach their conclusion that gender differences in doctoral students’ perceived university support of entrepreneurship leads to the gender gap in doctoral entrepreneurship.

First off, I commend the authors for pursuing this survey for a large, representative sample of Italian doctoral students. There is limited data on doctoral students and their preferences for entrepreneurship, so this survey will likely be of use for many topics of interest. As such, I expected more detail about the survey in this paper. In their submission, the authors reference another one of their papers that discussed the survey and provided a data sample; this information would have also been helpful in this paper for readers to get a full picture of what the survey entailed.

RESPONSE: Thank you for the encouraging words. We extended the description of the survey.

The authors also provided an original copy of the survey in Italian with their supporting materials; I would encourage submitting an English translation of the survey as well. This would better allow English-speaking readers to follow specific survey questions as they are referenced in the paper.

RESPONSE: We translated the questionnaire and attached it to the paper.

Giving the readers more information about the survey will allow them to better follow the paper’s analyses, which could be performed more rigorously and with more detail. As the analyses currently stand, the results provided are lacking information to reach the conclusions drawn in the paper. The strongest evidence provided are the t-tests for gender differences in perceptions of successful entrepreneurship, obstacles, and their parent university’s entrepreneurship environment (Tables 1-3). However, for these tables, the authors did not provide a p-value and rather used asterisks to denote the range of potential p-values. Providing the actual p-value or a standard error for these t-tests could better help readers determine the significance of these results.

RESPONSE: We added the standard error to the tests.

The authors then draw comparisons between the gender gap in entrepreneurship and entrepreneurial intention with students’ university environment. They again use a battery of t-tests; because they are drawing comparing across gender and across university characteristics, the t-tests are difficult to interpret as correlations between the two. It would be more appropriate to use a regression that interacts characteristics of the university environment with gender. The authors also describe analyses that provide different cuts of the data, such as focusing on only students with high entrepreneurial intention. However, their paper does not provide results with those separate cuts of the data; for example, Table 4 (the final table) appears to perform analyses for the entire dataset. This makes it difficult for readers to determine whether the authors’ conclusions are adequately supported by the data.

RESPONSE: We added an econometric analysis to the paper, on the whole sample as well as on two subsamples, one including men and one including women. The results support the conclusions in the paper. We omitted the descriptive statistics because of the strict requirements of the journal but they are available upon request.

Overall, Novo-Cortí & Muscio (2021) has the potential to be informative of the gender gap in doctoral entrepreneurship. In its current state, the paper requires more detail and additional analyses to solidify the conclusions about how university support differentially affects male and female students’ entrepreneurial tendencies. Thus, I recommend a major revision and resubmission.
